# Coronary MR Angiography in patients with coronary artery disease using image-based respiratory motion compensation

**DOI:** 10.1186/1532-429X-17-S1-P85

**Published:** 2015-02-03

**Authors:** Markus Henningsson, Konstantinos Bratis, Eike Nagel, Rene Botnar

**Affiliations:** 1 Division of Imaging Sciences and Biomedical Engineering, King's College London, London, UK

## Background

Respiratory motion remains a major impediment in a substantial amount of patients undergoing coronary magnetic resonance angiography (CMRA). Studies in healthy subjects have shown that image-based navigation (iNAV) improves respiratory motion compensation compared to the state-of-the art method. Here, we used iNAV for respiratory motion compensated CMRA in 22 patients with known or suspected coronary artery disease.

## Methods

The iNAV allowed for direct tracking of translational respiratory motion of the heart in 2D (foot-head and left-right motion) and was generated using the startup echoes of a bSSFP sequence. To improve robustness of this method, respiratory gating was implemented using the diminishing variance algorithm (DVA). In this approach k-space was completely filled during the first phase of the scan, while in the second phase the most motion corrupted data was discarded and re-acquired. If N shots were needed to fill the entire k-space without gating then both DVA phases one and two consisted of N shots, resulting in a 50% gating efficiency. Motion correction and DVA gating with iNAV for CMRA was integrated into the software of a clinical 1.5T scanner (Philips Healthcare, Best, Netherlands) and no post-processing was required. The whole-heart CMRA images were acquired with 1.3mm isotropic resolution and a SENSE factor of 2.5 in phase encoding direction. We compared the findings of the CMRA with iNAV against gold standard coronary X-ray or CT.

## Results

**Figure 1:**
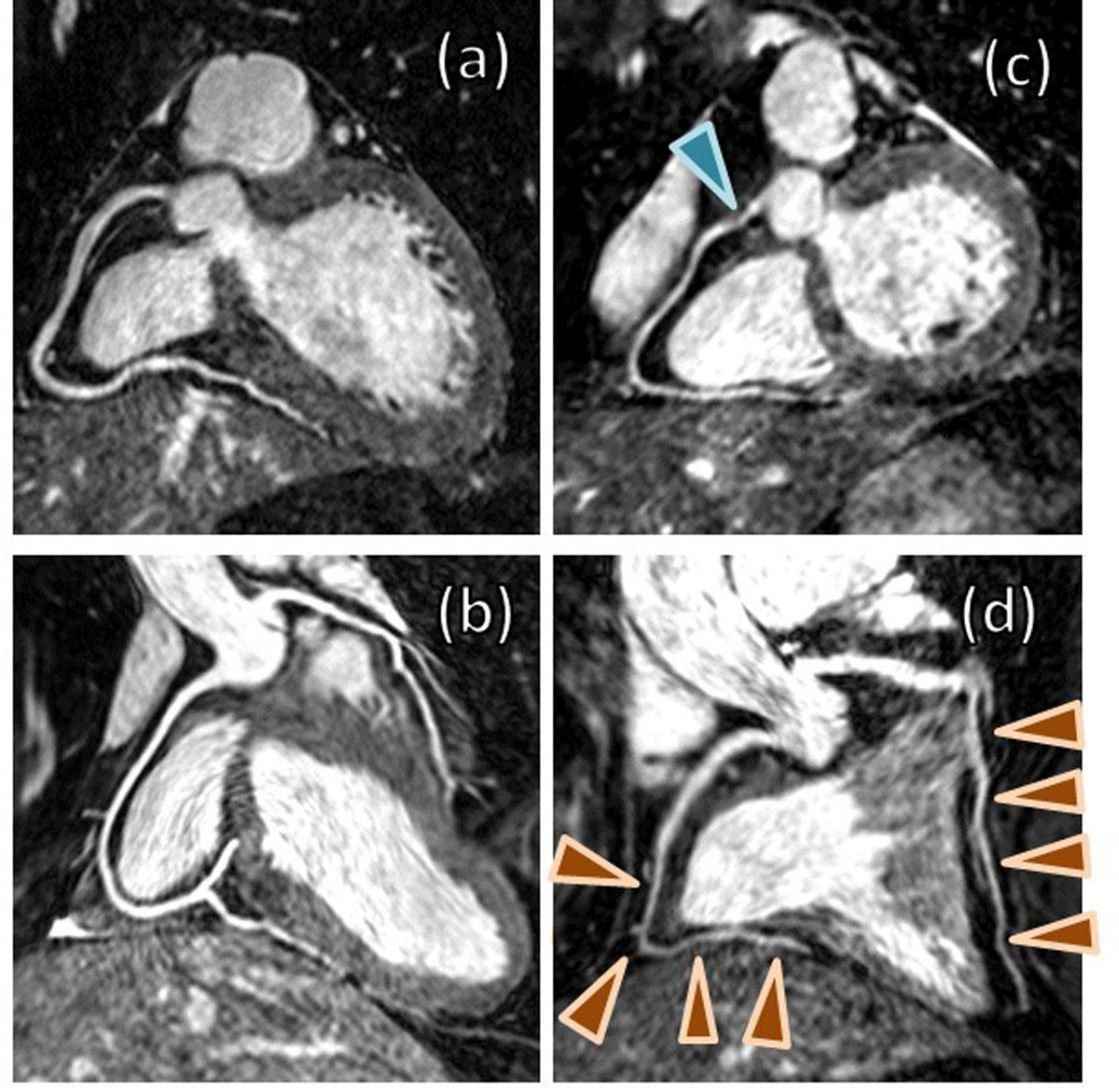
CMRA obtained using iNAV in four patients with suspected CAD: 58-year old female with no CAD (a), 37-year old male with no CAD (b), 60-year old male with a 75% stenosis in the proximal RCA (c), and 67-year old male with diffuse CAD in the mid and distal segments of the RCA and LAD (d).

In the 22 patients, 66 coronary vessels were analysed (right coronary artery, left anterior descending and left circumflex), and 11 vessels were found to be diseased (lumen diameter < 50%) in a total of 7 patients based on the reference X-ray or CT angiograms. CMRA scans with iNAV were successfully obtained in all patients with an average scan time of 7:40±0:29 min:sec. Representative images from four patients, two with no CAD and two with confirmed CAD, are shown in Figure 1. The final gating window using DVA gating was found to be 3.8±1.3 mm. The sensitivity and specificity of the proposed approach on a per vessel basis was 90% [confidence interval (CI): 59%-98%] and 100% (CI: 90%-100%) respectively, and on a per patient basis 86% (CI: 42%-98%) and 100% (CI: 78%-100%).

## Conclusions

The proposed iNAV method provides whole-heart CMRA in a short and predictable scan time while minimizing respiratory motion artifacts. Preliminary results of the proposed iNAV approach for CMRA indicate that it may provide diagnostic information approaching that of the gold standard methods which utilize ionizing radiation. However, due to the small patient number in this study further work is required to validate the clinical usefulness of CMRA using iNAV motion correction for patients with coronary artery disease. Improved diagnostic accuracy may be achieved by using vasodilating nitrates during the CMRA which will be incorporated in future studies.

## Funding

British Heart Foundation; Grand number: RG/12/1/29262.

